# *anti*-Tricyclo­[4.2.1.1^2,5^]deca-3,7-diene-9,10-dione

**DOI:** 10.1107/S1600536809005844

**Published:** 2009-03-11

**Authors:** Matthew P. Gidaly, Andria D. Harris, Maria del Rosario I. Amado-Sierra, Daniel S. Jones, Markus Etzkorn

**Affiliations:** a Department of Chemistry, The University of North Carolina at Charlotte, 9201 University City Blvd, Charlotte, NC 28223, USA

## Abstract

The title compound, C_10_H_8_O_2_, is a precursor to an unusual bis-homoaromatic dication and to heterodiamantanes and other oxa-cage compounds. Two independent mol­ecules, each of which is situated on a center of symmetry, comprise the unit cell. Both mol­ecules are in nearly identical chair conformations.

## Related literature

For related structures, see: Eaton *et al.* (2002 ▶); Harris *et al.* (2008 ▶); Masters *et al.* (1994 ▶). For the synthesis and related details, see: Hafner & Goliasch (1961 ▶); Weiss *et al.* (1960 ▶); Dilthey & Quint (1930 ▶); Garbisch & Sprecher (1966 ▶); Saito & Ito (2008 ▶); Baggiolini *et al.* (1967 ▶); Klinsmann *et al.* (1972 ▶); Amman *et al.* (1980 ▶); Amman & Ganter (1977 ▶, 1981 ▶); Prakash *et al.* (1987 ▶); Harris *et al.* (2008 ▶).
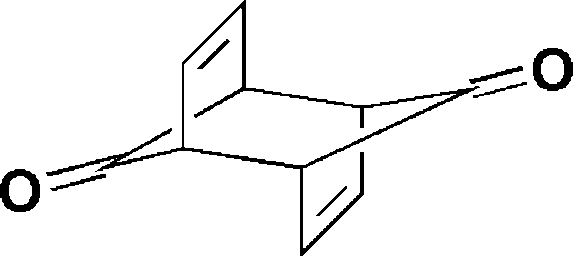


## Experimental

### 

#### Crystal data

C_10_H_8_O_2_
*M* *_r_* = 160.16
Triclinic, 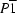

*a* = 6.4458 (7) Å
*b* = 6.6120 (6) Å
*c* = 8.9758 (6) Å
α = 81.671 (8)°
β = 79.176 (10)°
γ = 84.745 (8)°
*V* = 370.96 (6) Å^3^
*Z* = 2
Cu *K*α radiation
μ = 0.82 mm^−1^
*T* = 295 K
0.3 × 0.2 × 0.2 mm

#### Data collection

Enraf–Nonius CAD-4 diffractometer
Absorption correction: none
2624 measured reflections
1329 independent reflections
1179 reflections with *I* > 2σ(*I*)
*R*_int_ = 0.045
3 standard reflections every 82 reflections intensity decay: 1%

#### Refinement

*R*[*F* ^2^ > 2σ(*F* ^2^)] = 0.037
*wR*(*F* ^2^) = 0.092
*S* = 1.12
1329 reflections
110 parameters
H-atom parameters constrained
Δρ_max_ = 0.2 e Å^−3^
Δρ_min_ = −0.15 e Å^−3^

### 

Data collection: *CAD-4 EXPRESS* (Enraf–Nonius, 1994 ▶); cell refinement: *CAD-4 EXPRESS*; data reduction: *XCAD4* (Harms & Wocadlo, 1995 ▶); program(s) used to solve structure: *SHELXS97* (Sheldrick, 2008 ▶); program(s) used to refine structure: *SHELXL97* (Sheldrick, 2008 ▶); molecular graphics: *ORTEP-3 for Windows* (Farrugia, 1997 ▶); software used to prepare material for publication: *WinGX* (Farrugia, 1999 ▶).

## Supplementary Material

Crystal structure: contains datablocks global, I. DOI: 10.1107/S1600536809005844/fl2229sup1.cif

Structure factors: contains datablocks I. DOI: 10.1107/S1600536809005844/fl2229Isup2.hkl

Additional supplementary materials:  crystallographic information; 3D view; checkCIF report

## Figures and Tables

**Fig. 1.**
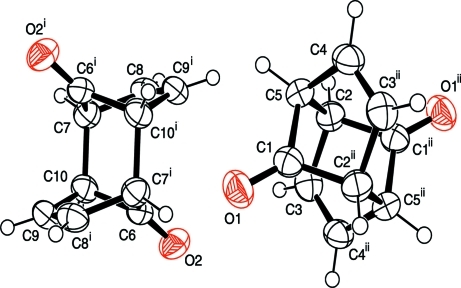
View of the two independent molecules of the title compound, 4, with 50% probability displacement ellipsoids. [Symmetry codes: (i) -x, -y + 1, -z + 2; (ii) -x + 1, -y + 2, -z + 1]

**Fig. 2.**
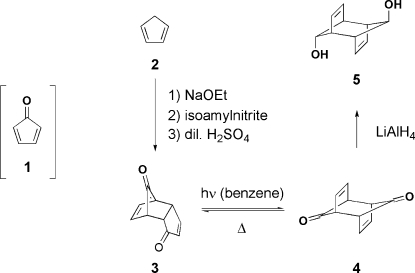
The formation of the title compound.

## supplementary crystallographic information

### Comment

The polycyclic title compound, dione 4, is formally a dimer of the elusive cyclopentadienone 1. The latter compound has only a fleeting existence and could be trapped in the form of its Diels-Alder adduct 3 (Hafner & Goliasch, 1961) or stabilized as an iron pentacarbonyl complex (Weiss *et al.*, 1960), although derivatives with bulky substituents have been prepared as stable monomers (Dilthey & Quint, 1930; Garbisch & Sprecher, 1966; Saito & Ito, 2008). The title compound 4 is accessible through photoisomerization of Diels-Alder adduct 3, a transformation that has been thoroughly studied (Baggiolini *et al.*, 1967; Klinsmann *et al.*, 1972) since dione 4 – *via* diol 5 (Amman *et al.*, 1980; Amman & Ganter, 1977; Amman & Ganter, 1981) - is a valuable precursor to an unusual bishomoaromatic dication (Prakash *et al.*, 1987) and to heterodiamantanes and other oxa-cage compounds (Amman *et al.*, 1980; Amman & Ganter, 1977; Amman & Ganter, 1981). We have recently reported the structure of diol derivative 5 (Harris *et al.*, 2008) and herein report the structure of the parent dione 4.

Two independent molecules, each of which is situated on a center of symmetry, comprise the unit cell. Both molecules are in nearly identical "chair" conformations, with a maximum deviation between corresponding bond lengths of 0.01 Å. The molecular packing exhibits several short intermolecular contacts, with the shortest being 0.15 Å less than the sum of the van der Waals radii.

Two related structures have been reported. The first (Eaton *et al.*, 2002) has a chlorine atom in place of each hydrogen atom of the title compound, while the second (Masters *et al.*, 1994) lacks the double bonds of the title compound and has methyl groups on each of the four bridgehead carbon atoms.

### Experimental

The synthesis of the title compound, 4, is described in our previous structure report (Harris *et al.*, 2008). Crystals for data collection were obtained from a chloroform solution.

### Refinement

H atoms were constrained using a riding model. The olefinic C—H bond lengths were fixed at 0.93 Å and the methine C—H bond lengths at 0.98 Å, with *U*_iso_(H) = 1.2 U_eq._ (C).

### Crystal data

**Table:**

C_10_H_8_O_2_	*Z* = 2
*M**_r_* = 160.16	*F*(000) = 168
Triclinic, *P*1	*D*_x_ = 1.434 Mg m^−^^3^
Hall symbol: -P 1	Cu *K*α radiation, λ = 1.54178 Å
*a* = 6.4458 (7) Å	Cell parameters from 24 reflections
*b* = 6.6120 (6) Å	θ = 10.1–44.8°
*c* = 8.9758 (6) Å	µ = 0.82 mm^−^^1^
α = 81.671 (8)°	*T* = 295 K
β = 79.176 (10)°	Prism, yellow
γ = 84.745 (8)°	0.3 × 0.2 × 0.2 mm
*V* = 370.96 (6) Å^3^	

### Data collection

**Table:**

Enraf–Nonius CAD-4 diffractometer	θ_max_ = 67.4°, θ_min_ = 5.1°
Non–profiled ω/2θ scans	*h* = −7→7
2624 measured reflections	*k* = −7→7
1329 independent reflections	*l* = −10→10
1179 reflections with *I* > 2σ(*I*)	3 standard reflections every 82 reflections
*R*_int_ = 0.045	intensity decay: 1%

### Refinement

**Table:**

Refinement on *F*^2^	H-atom parameters constrained
Least-squares matrix: full	*w* = 1/[σ^2^(*F*_o_^2^) + (0.0242*P*)^2^ + 0.0776*P*] where *P* = (*F*_o_^2^ + 2*F*_c_^2^)/3
*R*[*F*^2^ > 2σ(*F*^2^)] = 0.037	(Δ/σ)_max_ < 0.001
*wR*(*F*^2^) = 0.092	Δρ_max_ = 0.2 e Å^−^^3^
*S* = 1.12	Δρ_min_ = −0.15 e Å^−^^3^
1329 reflections	Extinction correction: *SHELXL97* (Sheldrick, 2008), Fc^*^=kFc[1+0.001xFc^2^λ^3^/sin(2θ)]^-1/4^
110 parameters	Extinction coefficient: 0.115 (5)
0 restraints	

### Special details

**Table:**

Geometry. All e.s.d.'s (except the e.s.d. in the dihedral angle between two l.s. planes) are estimated using the full covariance matrix. The cell e.s.d.'s are taken into account individually in the estimation of e.s.d.'s in distances, angles and torsion angles; correlations between e.s.d.'s in cell parameters are only used when they are defined by crystal symmetry. An approximate (isotropic) treatment of cell e.s.d.'s is used for estimating e.s.d.'s involving l.s. planes.

### Fractional atomic coordinates and isotropic or equivalent isotropic displacement parameters (Å^2^)

**Table:**

	*x*	*y*	*z*	*U*_iso_*/*U*_eq_	
O1	0.30420 (16)	0.67650 (15)	0.48107 (14)	0.0495 (3)	
O2	−0.21014 (18)	0.85668 (16)	0.94715 (16)	0.0593 (4)	
C5	0.5818 (2)	0.8181 (2)	0.58171 (17)	0.0369 (4)	
H5	0.5912	0.6967	0.6575	0.044*	
C10	−0.2032 (2)	0.5136 (2)	1.09484 (17)	0.0392 (4)	
H10	−0.3204	0.556	1.173	0.047*	
C8	0.1509 (2)	0.6126 (2)	1.13965 (18)	0.0438 (4)	
H8	0.1613	0.6898	1.2164	0.053*	
C7	0.0067 (2)	0.4394 (2)	1.15786 (17)	0.0409 (4)	
H7	−0.0169	0.3633	1.2611	0.049*	
C3	0.2728 (2)	1.0665 (2)	0.67086 (18)	0.0423 (4)	
H3	0.1766	1.0349	0.7606	0.051*	
C2	0.5100 (2)	1.0222 (2)	0.65388 (17)	0.0381 (4)	
H2	0.566	1.0299	0.747	0.046*	
C9	−0.2602 (2)	0.3614 (2)	1.00162 (19)	0.0427 (4)	
H9	−0.3583	0.2631	1.0381	0.051*	
C1	0.4297 (2)	0.8034 (2)	0.47378 (17)	0.0362 (4)	
C6	−0.1443 (2)	0.6803 (2)	0.96120 (18)	0.0395 (4)	
C4	0.7783 (2)	0.8433 (2)	0.46039 (19)	0.0420 (4)	
H4	0.9153	0.8016	0.4756	0.05*	

### Atomic displacement parameters (Å^2^)

**Table:**

	*U*^11^	*U*^22^	*U*^33^	*U*^12^	*U*^13^	*U*^23^
O1	0.0444 (6)	0.0450 (6)	0.0619 (8)	−0.0112 (5)	−0.0102 (5)	−0.0107 (5)
O2	0.0608 (7)	0.0390 (6)	0.0810 (9)	0.0080 (5)	−0.0283 (6)	−0.0043 (6)
C5	0.0368 (7)	0.0364 (7)	0.0373 (8)	0.0005 (5)	−0.0110 (6)	−0.0003 (6)
C10	0.0323 (6)	0.0466 (8)	0.0378 (8)	−0.0031 (6)	−0.0034 (5)	−0.0066 (6)
C8	0.0398 (7)	0.0495 (8)	0.0480 (9)	0.0004 (6)	−0.0172 (7)	−0.0161 (7)
C7	0.0418 (7)	0.0494 (8)	0.0317 (7)	−0.0050 (6)	−0.0099 (6)	0.0005 (6)
C3	0.0413 (7)	0.0427 (8)	0.0405 (8)	−0.0024 (6)	0.0024 (6)	−0.0102 (6)
C2	0.0421 (7)	0.0429 (8)	0.0312 (7)	−0.0024 (6)	−0.0106 (6)	−0.0060 (6)
C9	0.0335 (7)	0.0417 (8)	0.0554 (10)	−0.0060 (6)	−0.0128 (6)	−0.0057 (7)
C1	0.0331 (6)	0.0369 (7)	0.0395 (8)	0.0010 (5)	−0.0063 (6)	−0.0102 (6)
C6	0.0351 (7)	0.0397 (8)	0.0468 (9)	−0.0015 (6)	−0.0169 (6)	−0.0041 (6)
C4	0.0317 (7)	0.0420 (8)	0.0523 (9)	0.0026 (6)	−0.0083 (6)	−0.0086 (7)

### Geometric parameters (Å, °)

**Table:**

O1—C1	1.2049 (16)	C8—H8	0.93
O2—C6	1.2012 (17)	C7—C6^i^	1.522 (2)
C5—C4	1.5116 (19)	C7—H7	0.98
C5—C1	1.5202 (19)	C3—C4^ii^	1.326 (2)
C5—C2	1.575 (2)	C3—C2	1.5129 (19)
C5—H5	0.98	C3—H3	0.93
C10—C9	1.509 (2)	C2—H2	0.98
C10—C6	1.5232 (19)	C9—H9	0.93
C10—C7	1.5741 (19)	C1—C2^ii^	1.5248 (18)
C10—H10	0.98	C6—C7^i^	1.522 (2)
C8—C9^i^	1.326 (2)	C4—H4	0.93
C8—C7	1.512 (2)		
			
C4—C5—C1	96.67 (11)	C4^ii^—C3—C2	110.37 (12)
C4—C5—C2	111.33 (11)	C4^ii^—C3—H3	124.8
C1—C5—C2	105.88 (10)	C2—C3—H3	124.8
C4—C5—H5	113.8	C3—C2—C1^ii^	96.61 (10)
C1—C5—H5	113.8	C3—C2—C5	111.11 (11)
C2—C5—H5	113.8	C1^ii^—C2—C5	106.04 (11)
C9—C10—C6	96.32 (12)	C3—C2—H2	113.9
C9—C10—C7	111.22 (12)	C1^ii^—C2—H2	113.9
C6—C10—C7	106.35 (11)	C5—C2—H2	113.9
C9—C10—H10	113.8	C8^i^—C9—C10	110.46 (13)
C6—C10—H10	113.8	C8^i^—C9—H9	124.8
C7—C10—H10	113.8	C10—C9—H9	124.8
C9^i^—C8—C7	110.14 (14)	O1—C1—C5	128.74 (13)
C9^i^—C8—H8	124.9	O1—C1—C2^ii^	128.48 (14)
C7—C8—H8	124.9	C5—C1—C2^ii^	102.57 (11)
C8—C7—C6^i^	96.36 (11)	O2—C6—C7^i^	128.63 (14)
C8—C7—C10	111.33 (11)	O2—C6—C10	128.41 (15)
C6^i^—C7—C10	106.49 (12)	C7^i^—C6—C10	102.44 (11)
C8—C7—H7	113.7	C3^ii^—C4—C5	110.25 (12)
C6^i^—C7—H7	113.7	C3^ii^—C4—H4	124.9
C10—C7—H7	113.7	C5—C4—H4	124.9

Symmetry codes: (i) −*x*, −*y*+1, −*z*+2; (ii) −*x*+1, −*y*+2, −*z*+1.
